# Development of a Personalized Multiclass Classification Model to Detect Blood Pressure Variations Associated with Physical or Cognitive Workload

**DOI:** 10.3390/s24113697

**Published:** 2024-06-06

**Authors:** Andrea Valerio, Danilo Demarchi, Brendan O’Flynn, Paolo Motto Ros, Salvatore Tedesco

**Affiliations:** 1 Department of Electronics and Telecommunications, Politecnico di Torino, 10129 Torino, Italy; danilo.demarchi@polito.it; 2 Tyndall National Institute, University College Cork, Lee Maltings Complex, Dyke Parade, T12R5CP Cork, Ireland; brendan.oflynn@tyndall.ie (B.O.); salvatore.tedesco@tyndall.ie (S.T.)

**Keywords:** cuffless blood pressure, personalized health, photoplethysmogram, pulse transit time, pulse wave analysis

## Abstract

Comprehending the regulatory mechanisms influencing blood pressure control is pivotal for continuous monitoring of this parameter. Implementing a personalized machine learning model, utilizing data-driven features, presents an opportunity to facilitate tracking blood pressure fluctuations in various conditions. In this work, data-driven photoplethysmograph features extracted from the brachial and digital arteries of 28 healthy subjects were used to feed a random forest classifier in an attempt to develop a system capable of tracking blood pressure. We evaluated the behavior of this latter classifier according to the different sizes of the training set and degrees of personalization used. Aggregated accuracy, precision, recall, and *F*1-score were equal to 95.1%, 95.2%, 95%, and 95.4% when 30% of a target subject’s pulse waveforms were combined with five randomly selected source subjects available in the dataset. Experimental findings illustrated that incorporating a pre-training stage with data from different subjects made it viable to discern morphological distinctions in beat-to-beat pulse waveforms under conditions of cognitive or physical workload.

## 1. Introduction

Population growth and the aging demographic are recognized as predominant factors linked to the rise in the incidence of cardiovascular diseases (CVDs) [1]. The estimated increment in the number of adults aged 30 to 79 ranged from approximately 650 million to 1.28 billion between 1990 and 2019 [2,3]. Hypertension, also known as elevated blood pressure (BP), is a medical condition that promotes the insurgence of coronary diseases and different pathologies impacting vital organs, such as the brain and kidneys [4,5,6,7].

Noninvasive BP measurement systems have emerged to overcome the mentioned limitation of the invasive approach [8]. These devices employ multi-modal sensors that exploit diverse physical principles to extract information regarding the status of the cardiovascular system [9,10,11,12,13]. Continuous arterial BP measurement has been widely acknowledged as a more accurate determinant of cardiovascular risks since alterations in systolic or diastolic BP, along with changes in the shape of BP waveforms over time, reflect the progression of arterial and arteriolar modifications [14,15]. Moreover, by analyzing the arterial pressure waveforms, the cardiovascular status can be assessed through the estimation of physiological parameters [16,17]. A promising approach for continuous BP measurement is through computational modeling of the circulatory system [18,19,20]. These models integrate noninvasive data, like aortic flow and peripheral readings, to estimate BP. Parallelly, calibrated methods for cuffless BP, including Pulse Transit Time (PTT) and pulse wave analysis (PWA), stand out as viable solutions for BP assessment [21]. PTT is the time delay for the pressure wave to travel between proximal and distal arterial sites [22]. As defined in different arterial stiffness studies, PTT is conventionally identified as the foot-to-foot time delay between proximal and distal arterial BP waveforms [23,24]. PWA relies on extracting features from an arterial waveform and associating them with BP units through a calibration model. This approach offers greater convenience compared to the PTT method, as it necessitates only a single sensor [25] or can be employed in conjunction with PTT to enhance its accuracy. PWA employs data-driven feature extraction to extrapolate relevant information for the BP assessment. Numerous features have been investigated to analyze the morphology of the arterial pulse waves captured by photoplethysmography (PPG) sensors [26,27,28].

Several machine learning (ML) algorithms, including Support Vector Machines (SVM), random forests (RF), and feedforward neural networks (NN), have been employed in BP assessment [29,30,31]. In many instances, nonlinear models have demonstrated superior performance compared to linear models, although this outcome is contingent on the specific dataset and approach utilized, i.e., PTT, Pulse Arrival Time (PAT), or PWA using only PPG data. Additionally, more advanced methods, such as Recurrent Neural Networks (RNN) [32] and Long Short-Term Memory (LSTM) networks [33], have also been proposed. Although these models may offer a significant advantage over previously mentioned models by incorporating the ability to capture variations in extracted features over time, Deep Learning (DL) models require a large number of data samples to provide reasonably accurate BP values. Although DL and ML have found extensive application in BP assessment, the considerable inter-subject variability has posed challenges in formulating a sufficiently generalized model whose performance could also be maintained outside of the initial dataset. Therefore, drawing inspiration from established practices in the field of human activity recognition [34,35], numerous studies have suggested the formulation of person-specific models for the examination of this clinical parameter [36,37,38,39].

This paper presents a personalized ML model designed to detect BP changes in response to various stimuli. For this specific application, we developed a customized acquisition system to perform real-time acquisition and visualization of raw PPG pulse waveforms at the level of brachial (elbow) [10] and digital (thumb) [40] arteries. A specific data collection protocol was deployed to perform an accurate assessment and to induce a BP variation owing to the execution of both physical and cognitive tasks [41,42].

Our contributions in this application are as follows: (1) we propose ten pre-training strategies to calibrate an RF model based on individual physiological characteristics; (2) pre-training improves blood pressure classification accuracy on beat-to-beat pulse waveforms by up to 60% compared to a generalized model; (3) overfitting is mitigated by expanding the number of source subjects, reducing the need for additional target subject data; (4) we cut the data required to personalize the model by up to 30% while maintaining evaluation metrics above 95%. The structure of this article is as follows. Section 2 guides the reader through a detailed description of the hardware design of the device developed to retrieve the PPG raw data used in this work. Then, the data capture protocol and the data processing pipeline are presented, along with the description of the training strategies and the evaluation metrics employed to quantify the performance of the ML model. In Section 3, the results of the processing stage are reported, followed by the results retrieved for each subject in the dataset. Section 4 details the discussion and limitations of the proposed analysis. Finally, Section 5 concludes the paper.

## 2. Materials and Methods

### 2.1. Hardware Device

**Figure 1:**
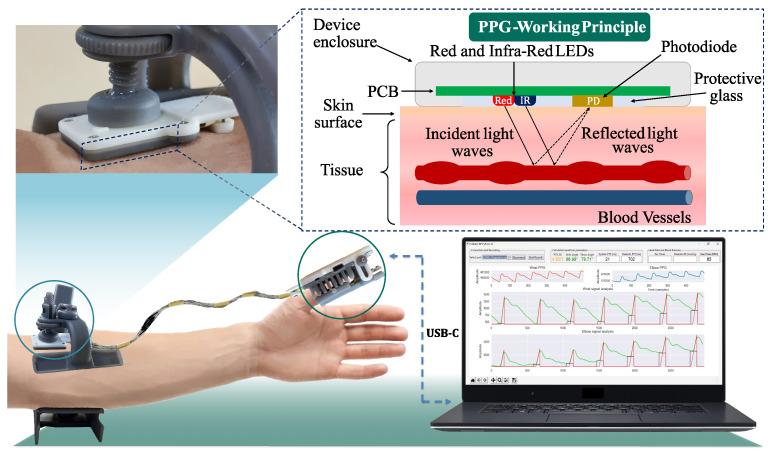
System employed to collect PPG raw data from the selected sites, elbow (brachial artery) and thumb (digital artery).

In this work, a custom-designed data acquisition system was employed to collect the arterial pulse waveforms between the brachial artery and the digital artery [43]. Figure 1 illustrates the fabricated supports and their positioning on the subject during data collection. The thumb-mounted holder (Figure 1, center) mimics a standard pulse oximeter design, featuring an elastic spring for sensor adherence to the finger. On the top left side of Figure 1, the sensor holder is seen to be positioned on the elbow, with mechanical fixtures that allow the operator to regulate arm pressure, making it steady during the data acquisition process as recommended in [21,44,45]. Each enclosure was designed to guarantee that the sensor maintains firm contact with the sample site, applying steady pressure and avoiding the need for the operator to hold it in its position. This feature enhances the replicability of the acquisition setup for any specific subject, thereby improving measurement consistency. Further information on the developed hardware device is available in [43].

### 2.2. Data Collection Protocol

A pre-clinical trial was carried out at the Tyndall National Institute in University College Cork (UCC), Cork, Ireland, to study the blood pressure variations related to the execution of cognitive and physical tasks. In this experiment, approved by the UCC Clinical Research Ethics Committee of the Cork Teaching Hospitals, a cohort of 31 healthy volunteers ranging from 21 to 34 years was recruited. Table 1 details the physiological parameters of the people involved. In accordance with the guidelines established for accurate BP measurement [46], every participant included in this study did not have any pre-existing cardiovascular condition and was not undergoing treatment with medications that could influence BP readings. Moreover, every individual was asked to refrain from smoking or consuming coffee in the 60 min before the session. The first step in the data capture consisted of the individual reclining in a supine position for 10 min to ensure that their hemodynamic conditions and vasomotor tone returned to a baseline level. Subsequently, the subject received instructions regarding the prescribed posture for data capture, which included sitting with back support, both feet flat on the floor, and hands resting on the table at a height equivalent to that of the heart.

**Figure 2:**
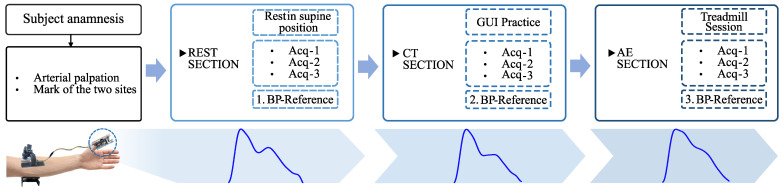
Data collection protocol followed in this study along with the evolution of the averaged pulse waveforms morphology according to each section of the data capture.

In accordance with the study protocol reported in Figure 2, after obtaining the anamnesis information, the operator identified the best location for the brachial artery through tactile arterial palpation. Once located, the spot was marked with ink to be sure that the acquisition site did not change over the duration of the data capture. Each data collection session was divided into three principal sections, denoted as follows: the resting phase (REST), the phase dedicated to cognitive testing (CT), and the after-exercise phase (AE). During each phase, a series of three data acquisitions, each one lasting one minute, was performed using the presented device. Then, the commercial cuff-based device BPM Connect [47] (Withings, Issy-les-Moulineaux, France) was used as a gold standard to measure the reference BP values for each specific section. In total, a set of three reference measurements was collected throughout the entire data collection. To prevent any potential recovery effects between measurements using both devices, we conducted the reference assessment immediately after completing the three acquisitions. Each phase was designed to induce changes in both blood pressure and PPG data collected from each participant. The resulting alterations in the PPG pulse waveforms are illustrated in the bottom section of Figure 2.

In the CT section, the subject was cognitively stimulated through two cognitive tests: the Stroop test [48] and the n-back test [49,50]. Both tests were executed through a custom-designed graphical user interface (GUI) structured to make a gradual augmentation in the level of complexity. Prior to commencing the actual measurement, the operator provided the participant with detailed instructions regarding the tests to be undertaken. Additionally, the participants had the opportunity to familiarize themselves with the GUI through the execution of a short demonstration. Then, the device was positioned on the subject. The last three minutes of this section were recorded during the execution of the n-back test and later subdivided into the three acquisitions related to the CT part. Hence, the reference BP measurement was taken again with the Withings device. Finally, the AE section of the data capture was performed. During this stage, each subject was engaged in a 10-min walking session on a calibrated treadmill. The treadmill’s configuration was kept uniform across all data collection sessions. The speed was configured at 8 km/h, and the inclination was adjusted to its maximum level to induce BP variation even in trained subjects. Then, the last three acquisitions with the proposed device were carried out along with the last reference BP measurement.

### 2.3. Data Processing

The data processing pipeline designed for this application can be divided into three major sections: pre-processing, pulse wave quality assessment (PWQA), and lastly, the identification of specific fiducial points employed to derive the features for data analysis. Thumb and elbow PPG measurements were processed following the same procedure within the MATLAB (Natick, MA, USA) environment. Each acquisition was band-pass filtered between 0.3 Hz and 15 Hz [51], to remove the DC offset and the high-frequency noise. Time series segmentation and labeling remain challenging areas of study, with researchers exploring various methods to improve accuracy and efficiency [52,53].

**Figure 3:**
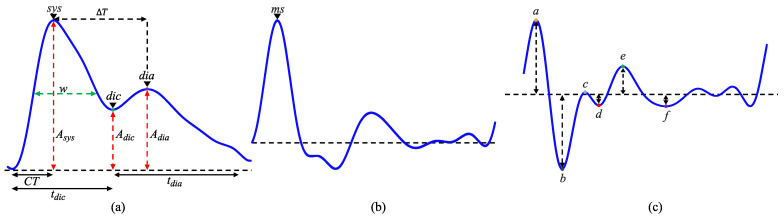
(**a**) Feature extracted from a PPG waveform. (**b**) Maximum of the first derivative (ms) detected on the velocity plethysmography (VPG). (**c**) Fiducial points detected on the acceleration plethysmography (APG).

This study addressed this challenge by segmenting our collected data into 3-s, consecutive, non-overlapping windows. This initial segmentation was used to identify and remove portions of the signal possibly corrupted by the presence of motion artifacts [54]. Subsequently, every single pulse wave within the acquisition was identified through the localization of the pulse onset, known in the literature as the beginning of the systolic phase within the cardiac cycle. The template matching approach was selected to perform the PWQA [55]. As the first step, a reference template was computed from all the available epochs. Then, Pearson’s correlation coefficient was used as the signal quality index (SQI) between the *i*th pulse and the template. All the pulses showing an indicator below the defined acceptance threshold (i.e., 0.95, 0.95, 0.9, respectively, for REST, CT, and AE) were marked as unacceptable and discarded. Feature measurements were obtained from the PPG pulse wave through the identification of key reference points on the pulse wave and its derivatives, which were then used to compute a variety of characteristics. The identified fiducial points included the systolic peak (sys), dicrotic notch (dic), and diastolic peak (dia) on the pulse wave, as well as the point of maximum upslope on the first derivative (ms). Additionally, the *a*, *b*, *c*, *d*, *e*, and *f* waves on the second derivative were detected [56,57,58,59]. These reference points are visually represented in Figure 3 for the baseline radial artery PPG pulse wave. Detailed criteria for identifying these fiducial points and features extracted are provided in Table 2 and Table 3, respectively.

### 2.4. Model Training

This study examined the efficacy of personalized against generalized training strategies to identify significant alterations in blood pressure levels. As delineated in section II-B, the data collection protocol was meticulously structured to induce BP variations through the execution of both physical and cognitive tasks. This setup enabled a thorough investigation of BP fluctuations in individuals subjected to diverse stimuli. In this context, a macroscopic variation in BP was defined as the difference between the reference values measured throughout the data collection procedure, regardless of the magnitude. Consequently, the phases of the data capture process (e.g., REST, CT, AE) were used as target labels for the analysis, as they inherently reflected BP changes.

**Figure 4:**
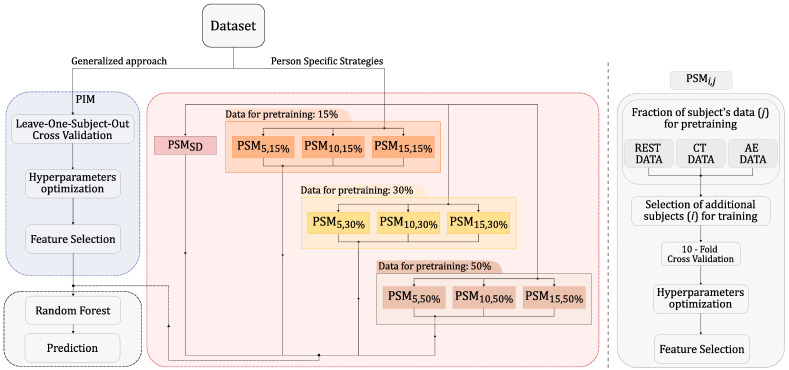
Overview of the tested training strategies. (Left) Workflow employed for the generalized approach (PIM). (Center) Tested combinations for person-specific strategies (PSMs). (Right) Workflow adopted by every $PSM_{i,j}$.

Our investigation compared the outcomes derived from applying ten different person-specific models (PSM) against those obtained by a person-independent model (PIM) when applied to the identical dataset, utilizing an RF classifier. Although features were extracted from signals at both sites, only those from the thumb were utilized. Pulse waves collected from the elbow were used to calculate the PTT, which was then used as a feature. Figure 4 (left branch) shows the workflow for our generalized approach. To optimize the model’s performance, we used a Leave-One-Subject-Out strategy across all users in the dataset. The optimization process involved the following parameters: the number of trees in each forest, which ranged from 50 to 100; the maximum depth of each tree in the forest, which ranged from 10 to 50; and the number of features used in the training process, which ranged from 3 to 25. The feature selection process was applied only at the training stages by ranking the first *n* features according to the mutual information between each feature and the target label. The right branch, highlighted in red, summarizes the ten personalized strategies.

The tested PSMs differed in the quantity of data used during the training phase and the fraction of the target subject data employed to personalize the model. Starting from $PSM_{SD}$, in which we used 50% of the data from the *k*th subject for training, data from randomly selected individuals were gradually added to the training set. Specifically, the number of source subjects varied across 5, 10, and 15 individuals. Different fractions of the target subject data were also tested to customize the model. This feature was progressively expanded, beginning from an initial value of 15%, and subsequently increased to 30% and 50%. Each combination of these parameters, when applied to the RF, was labeled as $PSM_{i,j}$ where *i* identifies the number of source individuals, *i* ∈ 5, 10, 15, and *j* refers to the percentage of data belonging to the *k*th target subject *j* ∈ 15%, 30%, 50%. The right side of Figure 4 details the workflow followed by each $PSM_{i,j}$ before applying the RF model. The initial step involved randomly selecting a portion of data samples from each class. To avoid class imbalance, we made sure that each class was equally represented by selecting 15%, 30%, or 50% of pulse waveforms from each class. Following this, pulse waveforms from different source subjects (5, 10, or 15) were included.

Then, unlike the generalized approach, the PSM method incorporated a 10-fold cross-validation to fine-tune the model’s hyperparameters and identify the most informative subset of features. Finally, all the mentioned solutions applied the RF model to predict the actual class of the input pulses. The output from each subgroup was merged for visualization purposes, although each PSM was tested individually.

### 2.5. Evaluation Metrics

As described in the previous subsection, the ten presented models were trained and tested for each subject in the dataset. Therefore, metrics such as accuracy, precision, recall, and *F*1-score were computed to evaluate the fluctuations in classification performance from subject to subject. Finally, an average of all indexes was computed along with its standard deviation to summarize the performance of each model. In a multiclass classification problem with three classes (REST, CT, and AE), the definitions are as follows:

- True positives (TP): correctly predicted instances of a class.
- False positives (FP): instances incorrectly predicted as a certain class.
- False negatives (FN): instances of a class that are incorrectly predicted as another class.
- True negatives (TN): all instances that are correctly not classified as the class under evaluation.

**Figure 5:**
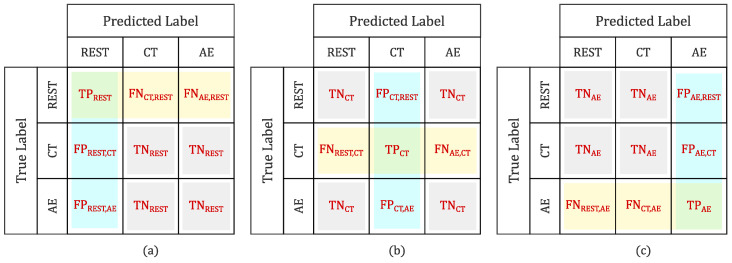
Definition of true positives (TP), false positives (FP), false negatives (FN), and true negatives (TN) instances in a multiclass problem. (**a**) REST class. (**b**) Cognitive task (CT) class. (**c**) After-exercise (AE) class.

Figure 5 clarifies how TP, FP, FN, and TN are identified respectively for each class.

The accuracy score, computed as the ratio of correctly predicted instances over of the total number of instances, was used to quantify the correctness of the predicted labels compared to the actual labels Equation (1).

(1) $$\mathrm{Accuracy}=\frac{\sum \mathrm{True}\mathrm{Positives}\mathrm{for}\mathrm{All}\mathrm{Classes}}{\mathrm{Total}\mathrm{Instances}}$$

Given these definitions, the evaluation metrics such as precision and recall scores were computed individually for each of the three classes referring to different sections of the data capture (α∈ REST, CT, AE) as specified in Equations (2) and (3).

(2) $$Precision_{\alpha }=\frac{TP_{\alpha }}{TP_{\alpha }+FP_{\alpha }}$$

(3) $$Recall_{\alpha }=\frac{TP_{\alpha }}{TP_{\alpha }+FN_{\alpha }}$$

where $FP_{\alpha }$ and $FN_{\alpha }$ are the overall numbers of false positives and false negatives referred to the target class α∈ REST, CT, and AE under evaluation.

Then, for every tested subject $sub_{i}$, the macro-averaged value of precision and recall, Equations (4) and (5), was calculated according to the following:

(4) $${\bar{Precision}}_{sub_{i}}=\frac{1}{N}\sum_{\alpha =1}^{N}Precision_{\alpha }$$

(5) $${\bar{Recall}}_{sub_{i}}=\frac{1}{N}\sum_{\alpha =1}^{N}Recall_{\alpha }$$

where *N* is the number of classes occurring in this study.

Finally, the the macro-averaged *F*1-score was computed as reported in Equation (6):

(6) $${\bar{F1}}_{sub_{i}}=\frac{1}{N}\sum_{\alpha =1}^{N}\frac{2\times Precision_{\alpha }\times Recall_{\alpha }}{Precision_{\alpha }+Recall_{\alpha }}$$

## 3. Results

### 3.1. Data Processing

Table 4 shows the results of the three processing stages described divided per section of the data capture (REST, CT, AE), for a single site.

After eliminating epochs corrupted by motion artifacts, the total number of segmented waveforms amounted to 19,274, distributed as follows: 6348 for the resting phase (REST), 6213 during cognitive testing (CT), and 6713 after the physical exercise phase (AE). Data collected from subject #26 were discarded due to corruption of the recording on both sites during the CT section. The variance in the number of detected waves aligned with the execution of the scheduled tasks during the data capture. Specifically, the approximately 400-wave difference between the REST section and the measurement following treadmill walking could be attributed to the observed increases in heart rate (HR) and BP in the measurements conducted with the reference device. Regarding the CT section, although there was an increase in SBP and DBP values (Table 5), the heart rate remained essentially constant compared with the resting value. This phenomenon was reflected in the number of waves detected (6213, CT vs. 6348, REST).

As a result of the PWQA, approximately 3.2% of the total pulses were excluded due to their insufficient similarity to the reference template. Due to the low data quality found in the CT section, data from subjects #17 and #29 were discarded from the dataset used for data analysis. Finally, following the validation of the fiducial points, an additional 4% of data points were discarded for a total of 17,886 pulse waves used in the analysis phase collected from 28 out of 31 subjects.

### 3.2. Model Evaluation

Table 6 compares the aggregated BP classification performance between ten different PSMs with the results achieved using a generalized approach. The results were expressed in terms of mean value and related standard deviation using the scoring criteria (accuracy, precision, recall, and *F*1-score) mentioned in Section 2.5. The evaluation metrics computed for each subject according to the training strategy are reported in Table A1, Table A2, Table A3, Table A4, Table A5, Table A6, Table A7 and Table A8 in Appendix A. The person-independent model, denoted as *PIM*, was trained across the complete dataset employing a Leave-One-Subject-Out cross-validation. Subsequently, performance evaluation was conducted by aggregating the outcomes obtained for each individual. No personalization was applied in this case. The low scores retrieved for each metric (0.36, 0.36, 0.31, and 0.37) suggest the requirement for personalization to model the PPG-BP relationship effectively.

**Figure 6:**
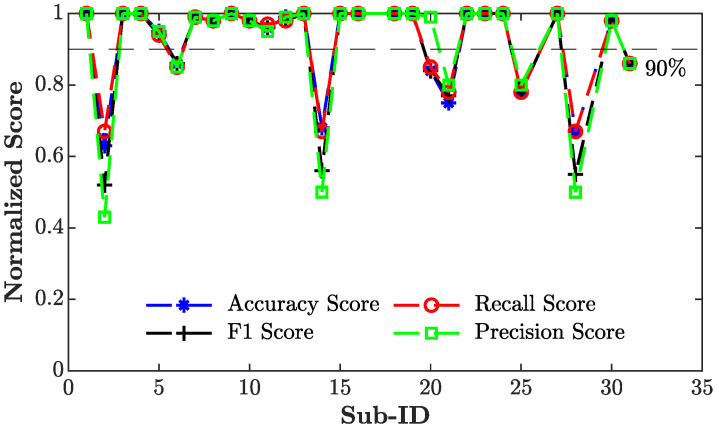
Values of evaluation metrics (accuracy, precision, recall, and *F*1-score) according to the training strategy denoted as $PSM_{SD}$. A 90% threshold (indicated by the gray dashed line) is used to identify subjects whose performance drops by more than 10% compared to the training phase.

**Figure 7:**
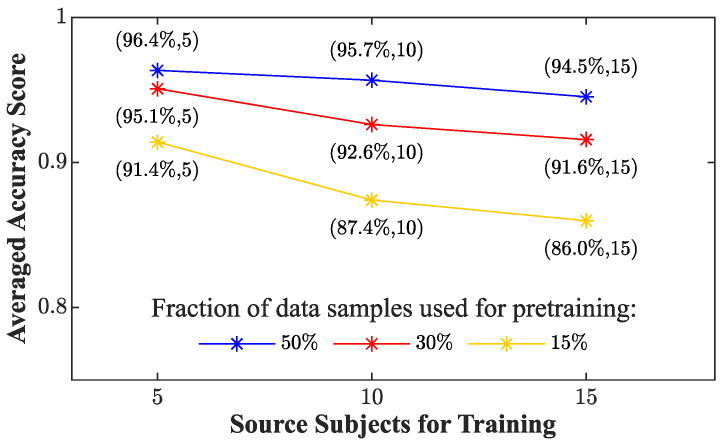
Averaged values of accuracy score according to different combinations of number of source subjects and diverse fractions of data employed to personalize the RF model.

Figure 6 displays the results gathered using the RF trained using 50% of the data of the target subject under investigation ($PSM_{SD}$). In this case, subjects #2, #14, #21, #25, and #28 displayed a marked decrease in classification performance, showcasing accuracy values of 0.63, 0.67, 0.75, 0.78, and 0.67, alongside precision values of 0.43, 0.5, 0.8, 0.8, and 0.5. Through a systematic assessment using the proposed training approaches, we examined how an alteration in the number of subjects and the percentage of data used for model customization impacted the classification performance. The evaluation metrics computed for each personalized model ($PSM_{i,j}$) are reported in Table 6, and the average accuracy score is represented in Figure 7.

In the latter figure, two discernible trends can be identified. Specifically, the average accuracy is directly correlated with the increase in the percentage of data utilized during the pre-training phase and inversely correlated with an augmentation in the number of subjects. The observed accuracy values of 96.4%, 95.7%, and 94.5% in the first set (e.g., $PSM_{5,50\%}$$PSM_{10,50\%}$$PSM_{15,50\%}$) declined to 95.1%, 92.6%, and 91.6% in the second set (e.g., $PSM_{5,30\%}$, $PSM_{10,30\%}$, $PSM_{15,30\%}$), and further decreased to 91.4%, 87.4%, and 86% in the third set (e.g., $PSM_{5,15\%}$, $PSM_{10,15\%}$, $PSM_{15,15\%}$). We excluded combinations that demonstrated overfitting across multiple subjects by discarding those with accuracy and *F*1 values below 0.95. Therefore, $PSM_{5,30\%}$, $PSM_{5,50\%}$, and $PSM_{10,50\%}$ were selected. Given the comparable overall performances across subjects for these combinations, our choice for the best combination was guided by a balance between performance metrics and the minimized data requirement for model customization. This led us to favor $PSM_{5,30\%}$.

## 4. Discussion

This study compared the performance of person-dependent and generalized models adopted to track BP macro-variations associated with physical or cognitive workload using a random forest classifier. This model was chosen due to its ability to handle the nonlinear relationships that exist between the extracted features and the variation in BP [57]. In other studies, RF outperformed other nonlinear models such as SVM adopting a nonlinear kernel and neural networks [60]. Moreover, RF is less prone to overfitting compared to the other two mentioned MLAs [29]. Generalized solutions often struggle with the high inter-subject variability within the dataset, making it challenging to develop a universally applicable model. The choice between personalized and universal models depends on the specific context and objectives of the problem being addressed.

Personalized models, finely tuned to individual users’ characteristics, take into account factors like age, gender, medical history, and lifestyle to provide more accurate and relevant predictions of BP. This tailored precision proves particularly crucial for individuals affected by complex health conditions or unique risk factors. Despite these advantages, the construction and maintenance of personalized models for each user pose challenges. This process can be resource-intensive, especially in the field of large-scale applications involving a significant number of subjects. Moreover, privacy and data protection concerns come to the forefront, as the development of personalized models often necessitates access to sensitive user data.

Generalized models, in contrast, are crafted to exhibit proficiency across a diverse spectrum of users without the need for individual customization. This inherent versatility makes them more scalable and simpler to implement, eliminating the necessity for tailoring to each user’s unique attributes. The cost-effectiveness and ease of maintenance associated with generalized models make them particularly advantageous for applications boasting a large user base. However, this broad approach comes with a trade-off since generalized models may fail to capture the distinctive characteristics and preferences of individual users. Consequently, the predictions generated by these models may result in a lack of accuracy compared to their personalized counterparts.

This phenomenon, highlighted in [61], is also reflected in our findings where the averaged metrics of the generalized approach (0.36, 0.36, 0.31, 0.37) underline the difficulties in defining a univocal representative model for subjects with different physiological characteristics. A hybrid approach combining personalized and universal models, as investigated in this study, may be beneficial for blood pressure monitoring. A universal model could be used as a baseline to provide initial predictions for all users, and personalized models could be applied to increase the model’s performance where personalization is deemed critical, such as users with complex health conditions or unique risk factors accommodating the inherent diversity in BP patterns among different subjects.

In [62], the authors used a transfer learning technique that personalized specific layers of a pre-trained network to improve the performance of PPG-based BP estimation, highlighting the influence of the number of data samples and source subjects used for training. Our analysis of the results shows that, on average, all the PSMs improved the performance of the generalized model regardless of the number of source subjects employed for training. Moreover, by observing the metrics displayed in Table 6, strategies including data obtained from different individuals demonstrated better performance in comparison to the model constructed exclusively using data from the tested subject ($PSM_{SD}$) where, as reported in Figure 6, the classification performance of eight subjects witnessed a substantial decline. Subject #2 emerged as the most adversely affected, exhibiting a notable drop of all metrics up to 0.63, 0.43, 0.67, and 0.52 for accuracy, precision, recall, and *F*1-score, respectively. These fluctuations in classification performance are a direct consequence of the phenomenon of overfitting whereby the model cannot correctly predict data that differ from the small training set available. To mitigate this issue, we included data from 5, 10, or 15 randomly selected subjects from the dataset in addition to diverse fractions of the target subject’s data (15%, 30%, 50%). In this way, we were able to evaluate the behavior of the model according to different sizes of the training set, degrees of personalization, and combinations of hyperparameters. Table 6 revealed a distinct inverse correlation between the classification metrics and the increase in the number of individuals. This diminishing pattern suggests the potential implications linked to the higher variability introduced by additional source subjects with respect to the initial quantity of data used to pre-train the model. Hence, this phenomenon may reduce random forest customization and consecutively lead to poorer classification performance for the target subject under evaluation. In fact, as depicted in Figure 7, $PSM_{5,15\%}$, $PSM_{10,15\%}$, and $PSM_{15,15\%}$ showed a more pronounced decrease in accuracy value as the number of subjects increased compared to the cases with 30% and 50% of the target subject’s data.

**Figure 8:**
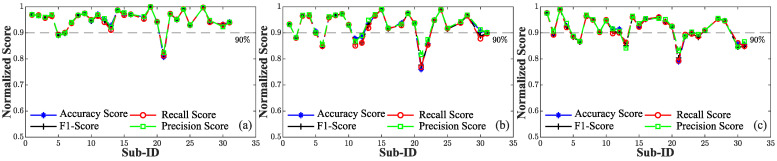
Evaluation metrics computed for each individual employing a fraction of the target subject data set equal to 30% and a diverse number of source subjects (N). A 90% threshold (indicated by the gray dashed line) is used to identify subjects whose performance drops by more than 10% compared to the training phase. (**a**) N = 5. (**b**) N = 10. (**c**) N = 15.

**Figure 9:**
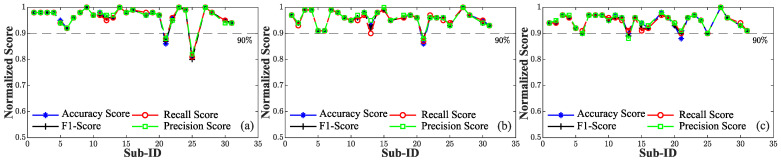
Evaluation metrics computed for each individual employing a fraction the target subject data set equal to 50% and a diverse number of source subjects (N). A 90% threshold (indicated by the gray dashed line) is used to identify subjects whose performance drops by more than 10% compared to the training phase. (**a**) N = 5. (**b**) N = 10. (**c**) N = 15.

This phenomenon is further visible in Figure 8 and Figure 9. Notably, when utilizing only 30% of the data for the pre-training stage, this adverse trend was further accentuated by a more pronounced variability (Figure 8b,c) compared to the scenario with 50% of the data (Figure 9b,c), where the standard deviation was progressively reduced. In the definition of the best solution within the context of our application, we opted to discard any tested combinations exhibiting aggregated accuracy and *F*1 values below 0.95. This approach ensured that combinations displaying overfitting across multiple subjects were not considered. As result, our selected PSMs were confined to $PSM_{5,30\%}$, $PSM_{5,50\%}$, and $PSM_{10,50\%}$. Upon analyzing the performance of various combinations across subjects within the dataset, it is evident that their overall performance values were generally comparable. However, an exception arose with subject #21, Figure 8a and Figure 9a,b, which exhibited a drop in performance exceeding 10% compared to the training phase in all three combinations, although slightly less evident in $PSM_{10,50\%}$. This trend was attributed to subject #21 having the lowest number of pulses in the dataset, resulting in a diminished dataset for personalization compared to other subjects. Notably, the combination $PSM_{15,50\%}$ demonstrated a substantial improvement, utilizing more data for personalization along with an increased number of individuals. Hence, due to the similarity observed among the performance metrics, the selection of the best combination was guided by the consideration of the data required for model customization, leading us to favor $PSM_{5,30\%}$. Employing 30% of the total available data, equivalent to approximately 162 s for the personalization phase, represents a noteworthy outcome. This achievement is particularly significant as it reflects a substantial reduction in the time required for this task compared to the approach outlined in [62], where 250 s of data recording per subject was used for the pre-training stage. Therefore, combining a subset of source subjects, in conjunction with an adequate fraction of data for pre-training leads to increased robustness and generalizability of personalized models across a broader spectrum of cases in BP assessment when compared to standard generalized models. Despite the mentioned improvements, some limitations of the proposed study need to be discussed. In this study, the performance of the proposed approach was evaluated on a limited sample of 28 subjects, falling short of the 85 subjects required by the AAMI [29]. To enhance model validation and generalization for accurate BP monitoring, it is crucial to include a diverse range of values that truly represent the population, including both males and females across different age ranges. In our future endeavors, we intend to extend the validation process to encompass a larger and more diverse cohort of individuals, aligning with the standards set by AAMI. Typically, to assess blood pressure variations, multiple sets of data collection over several days are conducted to ensure the algorithm’s consistent performance over time for the same individual. However, it is crucial to note that our data collection protocol was designed to induce short-term variations in BP linked to diverse stimuli rather than long-term monitoring. Moreover, increased proficiency in the cognitive tests section would likely result in reduced BP variation due to heightened familiarity with the tasks.

## 5. Conclusions

Cuffless blood pressure measurement has gained attention due to clinical demand and recent technological advances in the fields of data acquisition systems, embedded systems, and machine learning techniques. This paper presented a personalized multiclass classification model aimed at the detection of blood pressure variations associated with physical or cognitive workload. Several training strategies were implemented, each differing in the percentages of the dataset and utilizing a diverse subset of individuals as the training set. Experimental results demonstrated that the inclusion of a pre-training stage with data from diverse subjects enabled the discernment of morphological distinctions in beat-to-beat PPG waveforms under various stressors with respect to a generalized model fitted on the whole dataset. Understanding the regulatory mechanisms influencing blood pressure, combined with a reduction in the number of sensors employed to track this latter, constitutes a further step toward unobtrusive cuffless BP monitoring, resulting in better management of this parameter.

## Figures and Tables

**Table 1:** Overview of the characteristics of the study populations.

Characteristics	μ±σ	Range
Number of Subjects	31	-
Male	20 (64%)	-
Smokers	4 (13%)	-
Age (years)	27.77 ± 3.70	21–34
Height (cm)	172.74 ± 9.27	158–92
Weight (kg)	69.52 ± 12.72	53–99
BMI (kg m^−2^)	23.22 ± 3.30	18.16–31.24

Abbreviations: BMI, body mass index, μ, mean value, σ, standard deviation.

**Table 2:** Criteria for identifying fiducial points on PPG pulse waves.

Signal	Fiducial Point	Description
PPG, s	*Sys*	Maximum of the pulse waveform
	*Dic*	First local minimum after the systolic peak or coincident with e
	*Dia*	First local maximum after dic and before 0.8 T (where T is the duration of the cardiac cycle)
VPG, s′	*ms*	Maximum of the first derivative, s′
APG, s″	*a*	The maximum of s″ preceding the maximum of the first derivative ms
	*b*	First local minimum following a
	*c*	The greatest maximum of s″ between b and e (or, if no maxima, then the first maximum on x′ after e
	*d*	The lowest minimum on s″ after c and before e (or, if no minima, then coincident with c).
	*e*	The second maximum of s″ after ms and before 0.6 T (unless the c wave is an inflection point, in which case take the first maximum).
	*f*	The first local minimum of s″ after e and before 0.8 T.

Abbreviations: PPG, photoplethysmogram; VPG velocity plethysmography; APG, acceleration plethysmogram; s, original pulse; s′, first derivative of the original pulse; s″, second derivative of the original pulse.

**Table 3:** Definition of the extracted features from PPG pulse wave and its derivatives.

Signal	Type	Feature	Definition	Formula
PPG, s	Time	*ΔT*	Time delay between systolic and diastolic peaks	$t_{dia}-t_{sys}$
		*SI*	Stiffness index, h is the subject’s height	$h/(t_{dia}-t_{sys})$
		*CT*	Crest time: time occurring between pulse onset e of systolic peak	$t_{sys}-t_{0}$
		*w*	Pulse width at 50% of systolic peak amplitude, $A_{sys}$	-
		*IPR*	Instantaneous pulse rate	60/T
		*T*	Period of the cardiac cycle	-
		$t_{dia}$	Duration of the diastole	$T-t_{dic}$
		$t_{dic}$	Time to dicrotic notch	$t_{dic}-t_{0}$
	Amplitude	$A_{0}$	Amplitude of pulse onset	$s(t_{0})$
		$A_{sys}$	Amplitude of the systolic peak	$s(t_{sys})$
		$A_{dic}$	Amplitude of the dicrotic notch	$s(t_{dic})$
		$A_{dia}$	Amplitude of the diastolic peak	$s(t_{dia})$
		*RI*	Reflection index	$\left(s\left(t_{dia}\right)-s\left(t_{0}\right)\right)/\left(s\left(t_{sys}\right)-s\left(t_{0}\right)\right)$
		*K*	Pulse waveform characteristic value	$\left(s_{\mu }-A_{0}\right)/\left(A_{sys}-A_{0}\right)$
		$K_{1}$	Systolic characteristic value	$\left(s_{\mu ,sys}-A_{0}\right)/\left(A_{sys}-A_{0}\right)$
		$K_{2}$	Diastolic characteristic value	$\left(s_{\mu ,dia}-A_{0}\right)/\left(A_{sys}-A_{0}\right)$
		$s_{\mu ,sys}$	Mean value of the systolic phase of the pulse waveform	-
		$s_{\mu ,dia}$	Mean value of the diastolic phase of the pulse waveform	-
		$s_{\mu }$	Mean value of pulse waveform	-
		$s_{\sigma }$	Standard deviation of pulse waveform	-
		$s_{skewness}$	Skewness of pulse waveform	-
		$s_{kurtosis}$	Kurtosis of pulse waveform	-
	Area	$A_{1}$	Area under the curve between the pulse onset ($t_{0}$) and the dicrotic notch ($t_{dia}$)	-
		$A_{2}$	Area under the curve between the dicrotic notch ($t_{dia}$) and the end of the pulse ($t_{end}$)	-
		*IPA*	Inflection point area	$A_{2}/A_{1}$
VPG, s′	Time	$t_{ms}$	Time to the maximum slope computed on the first derivative of the pulse	$t_{ms}-t_{0}$
	Amplitude	$A_{ms}$	Amplitude of the maximum slope	$s^{\prime }\left(t_{ms}\right)$
APG, s″	Time	$t_{bd}$	Time elapsing between b and d	$t_{d}-t_{b}$
		$t_{bc}$	Time elapsing between b and c	$t_{d}-t_{c}$
	Amplitude	b/a	Amplitude ratio of early systolic negative wave over early systolic positive wave	$s^{\prime \prime }\left(t_{b}\right)/s^{\prime \prime }\left(t_{a}\right)$
		c/a	Amplitude ratio of late systolic re-increasing wave over early systolic positive wave	$s^{\prime \prime }\left(t_{c}\right)/s^{\prime \prime }\left(t_{a}\right)$
		d/a	Amplitude ratio of late systolic decreasing wave over early systolic positive wave	$s^{\prime \prime }\left(t_{d}\right)/s^{\prime \prime }\left(t_{a}\right)$
		e/a	Amplitude ratio of early diastolic positive wave over early systolic positive wave	$s^{\prime \prime }\left(t_{e}\right)/s^{\prime \prime }\left(t_{a}\right)$
		*AGI*	Aging index	$\left(s^{\prime \prime }\left(t_{b}\right)-s^{\prime \prime }\left(t_{c}\right)-s^{\prime \prime }\left(t_{d}\right)-s^{\prime \prime }\left(t_{e}\right)\right)/s^{\prime \prime }\left(t_{a}\right)$
Combined		*IPAD*	Inflection point area combined with d-peak	$A_{2}/A_{1}+s^{\prime \prime }\left(t_{d}\right)/s^{\prime \prime }\left(t_{a}\right)$
		*k*	Elasticity constant	$\frac{s^{\prime \prime }\left(t_{sys}\right)}{\left(\left(s\left(t_{sys}\right)-s\left(t_{ms}\right)\right)/\left(s\left(t_{sys}\right)-s\left(t_{0}\right)\right)\right)}$

**Table 4:** Data processing results.

Data Capture Phase	Segmented Pulses	PWQA	Fiducial Points Validation
REST	6348	6074	5935
CT	6213	5849	5630
AE	6713	6620	6321
Total Pulses	19,724	18,543 (96.8%)	17,886 (92.8%)

Abbreviations: REST, measurements at rest; CT, cognitive task section; AE, measurements after physical tasks; PWQA, pulse wave quality assessment.

**Table 5:** Averaged reference blood pressure values.

Data Capture Phase	SBP (mmHg)	DBP (mmHg)	HR (bpm)
REST	109 ± 11.6	67.8 ± 6.7	71.7 ± 8.3
CT	114.5 ± 3	71.2 ± 8.2	70 ± 8.7
AE	115.4 ± 12.2	72.9 ± 7.3	77.3 ± 11.9

REST, measurements at rest; CT, cognitive task section; AE, measurements after physical tasks; SBP, systolic blood pressure; DBP, diastolic blood pressure; HR, heart rate.

**Table 6:** Macro-averaged evaluation metrics computed for each model.

MLA	Training Strategy	Training Set				Test Set			
		Accuracy *	Precision *	Recall *	*F*1-Score *	Accuracy *	Precision *	Recall *	*F*1-Score *
RF	PIM	0.99	0.99	0.99	0.99	0.360 ± 0.200	0.360 ± 0.180	0.310 ± 0.180	0.370 ± 0.180
	${\mathrm{PSM}}_{SD}$	1	1	1	1	0.925 ± 0.117	0.912 ± 0.165	0.926 ± 0.113	0.912 ± 0.147
	${\mathrm{PSM}}_{5,50\%}$	1	1	1	1	0.964 ± 0.041	0.964 ± 0.040	0.963 ± 0.038	0.962 ± 0.042
	${\mathrm{PSM}}_{10,50\%}$	1	1	1	1	0.957 ± 0.030	0.958 ± 0.028	0.955 ± 0.030	0.956 ± 0.028
	${\mathrm{PSM}}_{15,50\%}$	1	1	1	1	0.945 ± 0.028	0.947 ± 0.028	0.945 ± 0.026	0.944 ± 0.027
	${\mathrm{PSM}}_{5,30\%}$	1	1	1	1	0.951 ± 0.039	0.952 ± 0.037	0.950 ± 0.038	0.950 ± 0.037
	${\mathrm{PSM}}_{10,30\%}$	1	1	1	1	0.926 ± 0.051	0.930 ± 0.044	0.922 ± 0.052	0.923 ± 0.051
	${\mathrm{PSM}}_{15,30\%}$	1	1	1	1	0.916 ± 0.045	0.918 ± 0.043	0.914 ± 0.045	0.914 ± 0.045
	${\mathrm{PSM}}_{5,15\%}$	1	1	1	1	0.914 ± 0.043	0.916 ± 0.043	0.913 ± 0.042	0.913 ± 0.042
	${\mathrm{PSM}}_{10,15\%}$	1	1	1	1	0.874 ± 0.069	0.885 ± 0.061	0.872 ± 0.072	0.871 ± 0.072
	${\mathrm{PSM}}_{15,15\%}$	1	1	1	1	0.860 ± 0.083	0.867 ± 0.083	0.852 ± 0.091	0.853 ± 0.091

Abbreviations: MLA, machine learning algorithm; PIM, person independent model; ${\mathrm{PSM}}_{SD}$, person-specific model with 50% of data from *k*th subject for training set, 25% as validation set, and 25% as test set; ${\mathrm{PSM}}_{i,j}$, person-specific model where *i* identifies the number of source individuals, *i*∈ 5, 10, 15, and *j* refers to the percentage of data belonging to the *k*th target subject *j*∈ 15%, 30%, 50%. Note: * macro-averaged values computed on the 28 subjects employed for the analysis.

## Data Availability

The data presented in this study are available on request from the authors.

## Appendix A

**Table A1:** Test set results using the person-independent model (PIM).

Sub-ID	Test Set				RF Hyperparameters	
	Precision	Recall	*F*1	Accuracy	Max Depth	Number of Estimators
1	0.35	0.36	0.28	0.35	20	80
2	0.19	0.22	0.20	0.23	20	80
3	0.38	0.29	0.19	0.28	20	80
4	0.19	0.27	0.19	0.27	20	80
5	0.54	0.36	0.32	0.34	20	80
6	0.43	0.54	0.44	0.51	20	80
7	0.74	0.74	0.74	0.75	20	80
8	0.36	0.40	0.35	0.39	20	80
9	0.35	0.40	0.36	0.42	20	80
10	0.09	0.27	0.14	0.26	20	80
11	0.41	0.45	0.40	0.53	20	80
12	0.64	0.60	0.60	0.64	20	80
13	0.16	0.16	0.16	0.19	20	80
14	0.77	0.59	0.53	0.60	20	80
15	0.15	0.33	0.21	0.44	20	80
16	0.40	0.19	0.16	0.18	20	80
17	-	-	-	-	-	-
18	0.30	0.30	0.29	0.30	20	80
19	0.09	0.20	0.12	0.20	20	80
20	0.38	0.45	0.38	0.43	20	80
21	0.36	0.27	0.20	0.29	20	80
22	0.18	0.10	0.11	0.10	20	80
23	0.09	0.20	0.13	0.21	20	80
24	0.46	0.54	0.49	0.56	20	80
25	0.56	0.63	0.52	0.63	20	80
26	-	-	-	-	-	-
27	0.15	0.34	0.20	0.35	20	80
28	0.72	0.60	0.53	0.59	20	80
29	-	-	-	-	-	-
30	0.25	0.30	0.20	0.35	20	80
31	0.13	0.24	0.14	0.23	20	80

**Table A2:** Test set results using person-specific model $PSM_{SD}$.

Sub-ID	Test Set				RF Hyperparameters	
	Precision	Recall	*F*1	Accuracy	Max Depth	Number of Estimators
1	1	1	1	1	50	60
2	0.43	0.67	0.52	0.63	10	60
3	1	1	1	1	10	60
4	1	1	1	1	10	60
5	0.95	0.94	0.94	0.95	10	90
6	0.85	0.85	0.86	0.86	50	100
7	0.99	0.99	0.99	0.99	10	60
8	0.98	0.98	0.98	0.98	10	60
9	1	1	1	1	10	60
10	0.98	0.98	0.98	0.98	10	60
11	0.95	0.97	0.96	0.96	10	60
12	0.99	0.98	0.98	0.99	10	90
13	1	1	1	1	20	70
14	0.5	0.67	0.56	0.68	10	60
15	1	1	1	1	10	60
16	1	1	1	1	10	60
17	-	-	-	-	-	-
18	1	1	1	1	10	60
19	1	1	1	1	10	80
20	0.99	0.85	0.84	0.84	10	70
21	0.8	0.78	0.77	0.75	20	60
22	1	1	1	1	10	100
23	1	1	1	1	50	60
24	1	1	1	1	10	60
25	0.8	0.78	0.78	0.78	10	60
26	-	-	-	-	-	-
27	1	1	1	1	10	60
28	0.50	0.67	0.55	0.67	10	90
29	-	-	-	-	-	-
30	0.98	0.98	0.98	0.98	30	60
31	0.86	0.86	0.86	0.86	10	70

**Table A3:** Test set results using person-specific model $PSM_{5,30\%}$.

Sub-ID	Test Set				RF Hyperparameters		
	Precision	Recall	*F*1	Accuracy	Max Depth	Number of Estimators	Subjects for Training
1	0.97	0.97	0.97	0.97	40	80	2 10 12 13 31
2	0.97	0.97	0.97	0.97	20	70	3 4 7 20 23
3	0.96	0.96	0.96	0.96	20	70	12 21 23 24 30
4	0.97	0.96	0.97	0.97	50	100	5 9 12 15 25
5	0.89	0.89	0.89	0.89	30	70	15 18 20 25 31
6	0.90	0.90	0.90	0.90	20	70	7 8 20 24 28
7	0.94	0.94	0.94	0.94	50	80	3 7 12 13 31
8	0.97	0.97	0.97	0.97	20	70	3 7 12 13 31
9	0.97	0.97	0.97	0.98	50	70	2 12 13 18 19
10	0.95	0.95	0.95	0.94	40	90	4 13 20 21 24
11	0.97	0.96	0.97	0.97	20	90	7 8 14 15 18
12	0.96	0.94	0.95	0.95	10	70	3 4 14 16 22
13	0.92	0.91	0.91	0.93	50	80	3 5 8 12 30
14	0.99	0.99	0.99	0.99	30	90	4 11 12 24 30
15	0.98	0.97	0.97	0.97	20	70	5 11 21 24 31
16	0.97	0.97	0.97	0.97	30	80	1 12 20 25 31
17	-	-	-	-	-	-	-
18	0.96	0.95	0.96	0.96	50	100	11 20 23 27 30
19	1.00	1.00	1.00	1.00	20	100	2 3 14 24 30
20	0.94	0.94	0.94	0.94	30	100	1 5 8 30 31
21	0.82	0.81	0.82	0.81	20	100	3 4 5 19 25
22	0.97	0.97	0.97	0.97	30	80	12 15 27 28 30
23	0.95	0.95	0.95	0.95	30	70	2 10 14 19 21
24	0.99	0.99	0.99	0.99	20	90	13 19 27 30 31
25	0.93	0.93	0.93	0.93	30	90	18 27 28 30 31
26	-	-	-	-	-	-	-
27	1.00	1.00	1.00	1.00	50	70	8 9 16 25 28
28	0.95	0.94	0.94	0.94	30	70	2 6 20 24 31
29	-	-	-	-	-	-	-
30	0.92	0.93	0.93	0.93	50	70	1 8 11 28 31
31	0.94	0.94	0.94	0.94	30	70	3 19 20 22 30

**Table A4:** Test set results using person-specific model $PSM_{10,30\%}$.

Sub-ID	Test Set				RF Hyperparameters		
	Precision	Recall	*F*1	Accuracy	Max Depth	Number of Estimators	Subjects for Training
1	0.93	0.93	0.93	0.93	20	100	2 7 8 12 13 14 15 20 22 31
2	0.88	0.88	0.88	0.88	40	90	1 3 7 15 18 21 22 23 28 31
3	0.97	0.97	0.97	0.97	40	90	2 8 9 11 12 13 16 20 25 27
4	0.97	0.96	0.97	0.97	30	80	1 2 10 15 16 21 22 23 27 31
5	0.90	0.90	0.90	0.91	40	100	2 4 6 9 21 22 23 27 28 30
6	0.86	0.85	0.85	0.85	50	80	4 5 7 9 13 19 21 22 27 31
7	0.96	0.96	0.96	0.96	30	100	3 4 5 8 10 14 15 20 22 31
8	0.97	0.97	0.97	0.97	40	90	4 5 6 9 12 13 14 16 23 27
9	0.97	0.97	0.97	0.97	50	100	1 3 4 14 15 18 21 23 24 25
10	0.93	0.93	0.93	0.93	20	100	6 9 12 14 15 16 22 25 28 30
11	0.87	0.85	0.86	0.88	30	100	3 10 12 18 21 23 24 27 28 30
12	0.89	0.86	0.86	0.89	50	100	1 4 6 7 11 18 19 21 23 31
13	0.95	0.92	0.93	0.93	20	90	2 3 6 10 11 14 15 18 20 28
14	0.97	0.97	0.97	0.97	30	80	4 7 9 11 12 16 19 20 23 25
15	0.99	0.99	0.99	0.99	30	100	1 2 4 11 13 20 24 25 27 31
16	0.92	0.92	0.92	0.92	50	100	6 10 12 13 15 19 25 27 28 30
17	-	-	-	-	-	-	-
18	0.93	0.93	0.93	0.94	50	70	1 5 6 10 14 20 21 27 28 30
19	0.98	0.98	0.98	0.98	50	70	6 9 10 14 20 22 23 25 27 28
20	0.94	0.94	0.94	0.94	50	90	1 2 6 7 8 11 19 21 27 31
21	0.82	0.77	0.78	0.76	30	90	2 5 6 10 14 16 22 24 25 31
22	0.88	0.85	0.85	0.86	40	80	4 5 6 7 10 12 18 24 25 30
23	0.95	0.95	0.95	0.95	40	70	8 9 16 19 20 21 24 25 28 31
24	0.99	0.99	0.99	0.99	30	100	1 4 6 8 10 11 14 22 27 30
25	0.92	0.92	0.91	0.92	50	100	1 3 4 12 15 19 21 22 28 31
26	-	-	-	-	-	-	-
27	0.94	0.94	0.94	0.94	40	90	6 7 9 14 16 18 24 28 30 31
28	0.97	0.97	0.97	0.97	40	100	1 4 8 13 14 18 19 23 24 25
29	-	-	-	-	-	-	-
30	0.91	0.88	0.89	0.91	30	90	1 4 6 10 13 18 22 25 27 28
31	0.90	0.90	0.90	0.90	50	80	2 5 10 15 16 19 20 23 24 30

**Table A5:** Test set results using person-specific model $PSM_{15,30\%}$.

Sub-ID	Test Set				RF Hyperparameters		
	Precision	Recall	*F*1	Accuracy	Max Depth	Number of Estimators	Subjects for Training
1	0.98	0.98	0.98	0.98	40	90	3 6 9 12 14 18 19 20 22 23 24 27 28 30 31
2	0.91	0.89	0.90	0.89	50	100	1 4 5 7 8 12 13 16 18 19 21 22 27 28 30
3	0.99	0.99	0.99	0.99	40	100	1 2 4 5 6 8 11 13 15 19 22 23 24 27 30
4	0.94	0.92	0.92	0.93	20	100	1 3 5 8 9 11 12 14 15 16 18 20 21 22 27
5	0.89	0.89	0.88	0.89	40	100	3 4 7 11 12 13 15 18 19 20 21 22 24 30 31
6	0.87	0.87	0.87	0.86	40	90	3 5 9 10 15 16 18 19 20 22 23 25 27 28 30
7	0.97	0.96	0.97	0.97	20	70	3 4 5 6 8 11 12 13 16 19 22 23 25 27 31
8	0.95	0.95	0.95	0.95	50	80	1 2 5 6 10 14 15 16 18 19 23 25 27 28 30
9	0.90	0.90	0.90	0.90	30	100	2 6 7 10 11 12 15 16 18 21 22 23 24 28 30
10	0.95	0.95	0.95	0.95	20	100	2 4 6 8 9 15 18 19 22 23 24 27 28 30 31
11	0.92	0.90	0.91	0.92	20	90	2 4 5 6 7 8 12 13 15 16 21 22 27 30 31
12	0.91	0.90	0.90	0.92	40	70	2 3 7 9 10 13 15 18 19 20 21 25 27 30 31
13	0.84	0.86	0.85	0.86	20	70	4 5 6 9 12 14 15 16 20 22 23 24 25 28 31
14	0.96	0.96	0.96	0.96	50	100	1 2 6 8 9 13 16 19 20 21 22 23 24 25 30
15	0.94	0.92	0.93	0.92	20	90	1 2 4 5 8 11 12 13 14 16 19 22 23 28 30
16	0.96	0.95	0.95	0.95	40	90	1 2 3 4 7 8 10 11 12 18 19 20 23 24 25
17	-	-	-	-	-	-	-
18	0.96	0.96	0.96	0.96	40	100	3 6 10 12 13 14 15 16 19 20 21 24 25 27 30
19	0.95	0.94	0.94	0.94	30	90	1 3 5 6 7 11 12 14 18 21 23 24 25 30 31
20	0.92	0.92	0.92	0.92	30	70	1 2 3 5 6 7 9 11 13 14 22 25 27 28 31
21	0.83	0.79	0.80	0.79	50	90	1 2 4 5 6 7 8 9 14 15 20 23 27 28 31
22	0.89	0.89	0.89	0.89	20	80	1 2 5 6 8 10 13 15 16 18 20 24 25 27 31
23	0.90	0.90	0.90	0.90	50	80	4 5 8 10 11 12 14 15 19 21 22 24 25 28 30
24	0.89	0.89	0.88	0.88	30	80	4 5 6 7 9 10 13 18 20 21 23 25 27 28 31
25	0.91	0.91	0.91	0.91	30	90	2 3 4 5 8 9 12 13 16 20 22 24 27 28 31
26	-	-	-	-	-	-	-
27	0.96	0.95	0.95	0.95	20	100	2 3 4 5 7 9 18 19 20 21 22 23 28 30 31
28	0.95	0.95	0.95	0.95	30	80	2 4 5 8 10 11 12 13 14 16 18 19 22 23 25
29	-	-	-	-	-	-	-
30	0.85	0.86	0.85	0.86	50	90	1 2 4 5 7 9 11 13 16 18 19 22 23 27 31
31	0.87	0.85	0.85	0.86	20	90	1 2 4 6 7 8 12 13 16 18 20 23 27 28 30

**Table A6:** Test set results using person-specific model $PSM_{5,50\%}$.

Sub-ID	Test Set				RF Hyperparameters		
	Precision	Recall	*F*1	Accuracy	Max Depth	Number of Estimators	Subjects for Training
1	0.98	0.98	0.98	0.98	30	80	10 14 16 27 31
2	0.98	0.98	0.98	0.98	20	60	8 9 22 24 25
3	0.98	0.98	0.98	0.98	20	100	9 10 16 21 25
4	0.98	0.98	0.98	0.98	40	100	13 20 22 30 31
5	0.94	0.94	0.94	0.95	40	80	7 15 22 28 31
6	0.92	0.92	0.92	0.92	50	80	7 8 15 21 22
7	0.96	0.96	0.96	0.96	20	100	1 15 19 28 30
8	0.98	0.98	0.98	0.98	50	70	4 12 20 23 27
9	1	1	1	1	20	90	5 6 8 24 25
10	0.97	0.97	0.97	0.97	20	80	8 9 11 19 23
11	0.98	0.97	0.97	0.98	20	100	2 10 15 19 23
12	0.97	0.95	0.96	0.96	20	60	2 4 15 16 25
13	0.97	0.96	0.96	0.96	30	80	6 10 11 21 27
14	1	1	1	1	40	90	3 7 20 21 27
15	0.98	0.98	0.98	0.98	20	100	6 10 12 16 27
16	0.99	0.99	0.99	0.99	30	70	4 5 9 10 13
17	-	-	-	-	-	-	-
18	0.97	0.98	0.97	0.97	30	90	10 11 14 20 31
19	0.98	0.98	0.98	0.98	20	100	5 6 8 10 13
20	0.97	0.97	0.97	0.97	30	80	6 8 22 23 28
21	0.88	0.88	0.87	0.86	50	80	4 12 14 24 31
22	0.95	0.96	0.95	0.96	50	80	7 15 16 22 28
23	1	1	1	1	40	90	9 11 19 22 27
24	0.99	0.99	0.99	0.99	40	90	7 11 16 18 19
25	0.82	0.81	0.80	0.81	20	80	1 14 15 19 22
26	-	-	-	-	-	-	-
27	1	1	1	1	30	90	1 7 9 12 28
28	0.98	0.98	0.98	0.98	30	70	5 12 21 22 27
29	-	-	-	-	-	-	-
30	0.94	0.95	0.95	0.95	30	60	1 2 5 21 23
31	0.94	0.94	0.94	0.94	40	80	7 22 23 25 30

**Table A7:** Test set results using person-specific model $PSM_{10,50\%}$.

Sub-ID	Test Set				RF Hyperparameters		
	Precision	Recall	*F*1	Accuracy	Max Depth	Number of Estimators	Subjects for Training
1	0.97	0.97	0.97	0.97	20	70	2 4 5 11 14 18 19 22 25 27
2	0.94	0.93	0.94	0.94	30	90	1 4 12 16 18 21 22 23 24 28
3	0.99	0.99	0.99	0.99	30	90	1 2 8 12 16 19 21 25 28 30
4	0.99	0.99	0.99	0.99	30	80	8 9 11 12 13 15 18 20 23 30
5	0.91	0.91	0.91	0.91	20	80	7 9 11 20 21 23 24 28 30 31
6	0.91	0.91	0.91	0.91	20	90	2 9 15 19 22 23 27 28 30 31
7	0.99	0.99	0.99	0.99	40	60	3 5 12 13 21 22 23 25 27 30
8	0.98	0.98	0.98	0.98	20	100	1 5 10 11 12 14 18 19 28 31
9	0.96	0.96	0.96	0.96	40	100	2 3 5 14 15 19 21 23 24 28
10	0.95	0.95	0.95	0.95	30	70	2 9 12 15 19 23 24 25 28 30
11	0.97	0.95	0.96	0.96	40	90	3 5 8 10 13 21 25 27 28 30
12	0.98	0.97	0.98	0.98	40	60	1 2 5 6 8 20 21 23 27 31
13	0.95	0.9	0.92	0.93	30	100	3 6 11 15 16 19 21 23 24 28
14	0.98	0.98	0.98	0.98	50	100	6 10 15 18 20 23 25 27 28 30
15	1	0.99	0.99	0.99	30	100	4 6 8 9 10 13 14 16 19 24
16	0.95	0.95	0.95	0.95	20	70	1 4 9 12 15 20 22 23 25 27
17	-	-	-	-	-	-	-
18	0.97	0.96	0.96	0.96	30	90	1 2 6 7 10 13 15 16 21 24
19	0.97	0.97	0.97	0.97	20	100	3 5 6 12 16 18 21 24 25 28
20	0.96	0.96	0.96	0.96	30	80	2 3 5 9 12 15 18 22 24 25
21	0.88	0.87	0.88	0.86	50	100	1 2 3 5 6 8 10 11 15 16
22	0.96	0.97	0.96	0.96	30	90	2 6 8 13 20 21 23 24 27 30
23	0.96	0.96	0.96	0.96	30	90	7 9 11 12 15 19 22 27 28 30
24	0.96	0.95	0.96	0.96	20	100	4 6 7 13 14 18 19 20 27 30
25	0.93	0.94	0.93	0.93	40	60	1 2 3 10 12 14 18 23 28 31
26	-	-	-	-	-	-	-
27	1	1	1	1	40	70	4 5 16 18 20 21 23 25 30 31
28	0.97	0.97	0.97	0.97	30	90	2 3 6 7 10 15 16 18 21 30
29	-	-	-	-	-	-	-
30	0.94	0.95	0.94	0.95	30	90	5 7 9 10 11 21 22 25 27 28
31	0.93	0.93	0.93	0.93	20	90	1 3 9 10 11 12 18 21 22 30

**Table A8:** Test set results using person-specific model $PSM_{15,50\%}$.

Sub-ID	Test Set				RF Hyperparameters		
	Precision	Recall	*F*1	Accuracy	Max Depth	Number of Estimators	Subjects for Training
1	0.94	0.94	0.94	0.94	50	90	2 3 5 6 9 10 13 15 16 19 22 23 25 27 31
2	0.95	0.94	0.94	0.94	20	100	3 4 5 9 12 13 14 15 16 18 19 20 21 25 30
3	0.97	0.97	0.97	0.97	20	100	1 2 5 9 10 11 12 15 19 21 22 23 24 25 30
4	0.97	0.96	0.96	0.96	50	80	2 3 7 8 10 12 14 16 18 21 22 23 27 28 30
5	0.92	0.92	0.92	0.92	20	90	1 3 10 12 13 14 15 16 18 19 20 24 25 27 28
6	0.9	0.91	0.9	0.9	30	100	1 3 5 7 8 9 11 12 13 16 20 22 25 27 28
7	0.97	0.97	0.97	0.97	20	80	1 2 4 9 10 13 14 16 21 22 23 24 28 30 31
8	0.97	0.97	0.97	0.97	50	80	2 5 7 10 13 14 18 19 21 22 23 25 27 28 31
9	0.97	0.97	0.97	0.97	50	90	2 5 6 7 8 13 14 15 16 19 20 21 23 30 31
10	0.95	0.96	0.95	0.95	20	100	2 3 8 13 14 15 18 21 22 23 24 25 27 28 30
11	0.97	0.96	0.96	0.97	20	80	2 3 7 9 10 12 13 14 15 16 20 22 24 27 28
12	0.96	0.95	0.96	0.96	30	90	3 5 6 8 10 11 18 19 20 22 23 25 27 30 31
13	0.88	0.91	0.89	0.9	30	90	1 2 8 9 10 11 14 15 19 20 21 22 23 25 30
14	0.96	0.96	0.96	0.96	50	90	1 4 6 9 10 11 12 15 16 18 20 23 24 28 31
15	0.98	0.98	0.98	0.98	20	60	1 2 7 9 12 13 14 20 21 22 23 24 25 27 28
16	0.93	0.92	0.92	0.92	40	80	4 5 8 9 10 12 18 20 21 22 25 28 27 30 31
17	-	-	-	-	-	-	-
18	0.98	0.97	0.97	0.98	30	90	1 3 6 12 13 14 15 16 19 21 22 23 27 28 31
19	0.96	0.96	0.96	0.96	40	100	1 3 4 5 8 10 11 12 13 18 20 21 22 25 27
20	0.94	0.94	0.93	0.93	50	100	2 5 7 8 9 11 12 14 16 18 22 23 25 27 28
21	0.91	0.9	0.9	0.88	20	100	2 4 5 9 10 13 14 16 18 19 20 23 24 27 28
22	0.96	0.96	0.96	0.96	40	100	4 6 7 8 11 13 14 15 19 20 21 23 27 28 30
23	0.97	0.97	0.97	0.97	20	100	1 2 8 9 11 12 14 19 20 21 22 24 27 28 30
24	0.95	0.95	0.95	0.95	40	90	2 3 4 6 8 11 14 16 18 20 21 23 28 30 31
25	0.9	0.9	0.9	0.9	30	80	1 2 3 5 6 7 9 14 16 18 19 21 24 27 28
26	-	-	-	-	-	-	-
27	1	1	1	1	20	90	2 4 8 13 14 15 16 18 19 20 21 23 24 28 30
28	0.96	0.96	0.96	0.96	20	80	2 5 7 8 9 11 12 14 16 18 19 23 24 28 30
29	-	-	-	-	-	-	-
30	0.93	0.94	0.93	0.93	30	90	2 5 8 9 10 11 12 14 16 20 21 22 25 28 31
31	0.91	0.91	0.91	0.91	30	90	1 2 3 6 10 11 12 13 14 15 21 22 25 28 30
